# Sleep Bruxism and Occurrence of Temporomandibular Disorders-Related Pain: A Polysomnographic Study

**DOI:** 10.3389/fneur.2019.00168

**Published:** 2019-03-11

**Authors:** Joanna Smardz, Helena Martynowicz, Monika Michalek-Zrabkowska, Anna Wojakowska, Grzegorz Mazur, Efraim Winocur, Mieszko Wieckiewicz

**Affiliations:** ^1^Department of Experimental Dentistry, Wroclaw Medical University, Wroclaw, Poland; ^2^Department of Internal Medicine, Occupational Diseases, Hypertension and Clinical Oncology, Wroclaw Medical University, Wroclaw, Poland; ^3^Department of Oral Rehabilitation, The Maurice and Gabriela Goldschleger School of Dental Medicine, Tel Aviv University, Tel Aviv, Israel

**Keywords:** sleep bruxism, pain, temporomandibular disorders, polysomnography, TMD pain screener

## Abstract

**Introduction:** The diagnosis of sleep bruxism is challenging due to the difficulties involved. Sleep bruxism can lead to clinical consequences, including pain in masticatory muscles, limitation of jaw mobility, headache, and the spectrum of symptoms associated with damage to the teeth and oral mucosa. Currently, only video-polysomnography can definitely diagnose sleep bruxism. Due to the risk of painful temporomandibular disorders (TMD) in sleep bruxers, early diagnosis of pain in the temporomandibular region using questionnaires is recommended. Therefore, this study aimed to assess the relationship between the intensity of sleep bruxism and the occurrence of pain related to TMD.

**Materials and Methods:** This study was conducted on the patients of the Clinic of Prosthetic Dentistry operating at the Department of Prosthetic Dentistry at the Wroclaw Medical University. Based on a positive medical history, a thorough examination for the diagnosis of probable sleep bruxism was carried out in the enrolled patients. Eligible patients were then subjected to a video-polysomnographic study. Each patient was asked to complete the TMD Pain Screener questionnaire to assess the occurrence of pain in jaw and temple area.

**Results:** The results of the study showed that increased bruxism episode index (BEI) was statistically significantly correlated with increase of all types of bruxism episodes—phasic, tonic, and mixed—in all the studied patients; a significant correlation was also found with respect to division of patients into studied and control groups. The study also showed that there was no statistically significant difference between BEI values and scores of TMD Pain Screener. In all the studied patients, a higher BEI was not found to be correlated with the occurrence of TMD-related pain assessed by TMD Pain Screener; similarly, no correlation was found with respect to division of patients into studied and control groups.

**Conclusions:** The occurrence of TMD-related pain is not related to the intensity of sleep bruxism. TMD Pain Screener may be used as an auxiliary tool in the diagnosis or risk of occurrence of TMD-related pain, whereas in the case of sleep bruxism, it has only limited diagnostic value.

**Clinical Trial Registration:** www.ClinicalTrials.gov, identifier NCT03083405

## Introduction

Bruxism is a common phenomenon, and is estimated to occur in 8−31% of the population without division into subtypes and without significant variation in relation to gender (1). Awake bruxism (AB) occurs in 22–31% and sleep bruxism (SB) in approximately 13% of adults (1, 2). The occurrence of bruxism decreases with age. Currently, in adults, bruxism is not considered as a disorder, but as a risk factor for other clinical consequences (1–5). In children, the unambiguous classification of symptoms commonly associated with bruxism is difficult. While bruxism occurring during sleep may be a physiological element of the natural maturation of the central nervous system, it may also be considered as a response to excessive stress, be caused by certain independent psychological and social factors, or constitute a protective mechanism in patients with sleep disorders (2, 3, 6, 7).

The origin of bruxism is multifactorial (1–3). It is now believed that bruxism may be caused by three groups of factors. The first group are biological factors, which include neurotransmitters, in particular dopamine, genetic factors, and cortical arousals. The second group are psychological factors, which include sensitivity to stress, individual character traits, and anxiety, among others. Both adults and children with bruxism have been shown to present higher scores on scales examining the intensity of stress, anxiety, and mental disorders compared to the control group. The third and a more popular group of factors causing bruxism are those of exogenous origin: nicotine, caffeine, alcohol, drugs, and some medications (3). There are also scientific reports indicating the comorbidity of bruxism with systemic disorders, including thyroid diseases, disorders of the digestive system, sleep disorders, and cardiovascular diseases (1–3, 6–8).

The widely accepted definition of bruxism was created in 2013 in an international consensus (1). Bruxism is defined as a repetitive activity of the jaw muscles, characterized by clenching or grinding of the teeth and/or bracing or thrusting of the mandible. This activity of jaw muscles may appear while wake (awake bruxism—AB) or during sleep (sleep bruxism—SB) (1, 3). The above definition formed the basis for the description of bruxism in the fourth edition of the guidelines for the evaluation, diagnosis, and treatment of orofacial pain of the American Academy of Orofacial Pain (2, 8) and the third edition of the International Classification of Sleep Disorders (2, 6). Although this definition was quickly and widely accepted due to its pragmatism, according to the latest suggestions, it requires verification, considering the hypothesis that bruxism occurring during wakefulness and the one occurring during sleep are separate phenomena and therefore require separate definitions (2). In 2018, as a part of the next international consensus, Lobbezoo et al. (2) proposed two separate definitions. According to these authors, SB can be defined as the activity of masticatory muscles during sleep, which can be rhythmic (phasic) or nonrhythmic (tonic), and should not be considered as a movement disorder or a sleep disorder in otherwise healthy individuals. On the other hand, AB is defined as an activity of the masticatory muscles during wakefulness which is characterized by repetitive or sustained tooth contact and/or by bracing or thrusting of the mandible and should not be considered as a movement disorder in otherwise healthy individuals. In both the definitions, the key phrase is “activity of the masticatory muscles,” indicating the potential clinical consequences of both the types of bruxism (2). Currently, bruxism is not considered as a disorder but referred to as a behavior that can act as a risk factor for detrimental disorders, or in contrast a protective factor for others (2, 7, 9).

Due to the difficulties involved, diagnosis of SB is challenging. SB can lead to clinical consequences, including pain in the masticatory muscles, limitation of jaw mobility, orofacial pain, headache in the temporal region, and the spectrum of symptoms associated with damage to the teeth and oral mucosa. Currently, only video-polysomnography can efficiently diagnose SB. According to the guidelines of the International Classification of Diseases-10-Clinical Modification (ICD-10-CM), during sleep, repeated contractions of the masticatory muscles occur in patients with SB are called as a rhythmic masticatory muscle activity (10). During electromyographic recording, these contractions can manifest as a series of repetitive activities (phasic contractions) lasting for 0.25–2 s or as isolated, long-lasting jaw clenching (tonic contractions) lasting over 2 s. A third type of contractions is also observed, which is a combination of phasic and tonic contractions, called mixed contractions (10, 11).

Due to the risk of pain related to temporomandibular disorders (TMD) in sleep bruxers, early diagnosis of pain in the temple and jaw area using questionnaires is recommended. One of the questionnaires used for diagnosis is TMD Pain Screener (12). This study aimed to assess the relationship between the intensity of SB and occurrence of pain related to TMD using the TMD Pain Screener.

## Materials and Methods

### Participants

The study population consisted of patients of the Prosthetic Dentistry Clinic operating at the Department of Prosthetic Dentistry at the Wroclaw Medical University. Based on a positive medical history and thorough examination following the guidelines of the ICD-10-CM of the American Academy of Sleep Medicine, probable SB was diagnosed in the patients.

This study was approved by the Ethical Committee of the Wroclaw Medical University (ID KB-195/2017). Written informed consent was obtained from all the participants of this study.

### Selection of Participants for Video-Polysomnography

The selection of patients for video-polysomnography was based on a medical interview with particular emphasis on the presence of severe systemic diseases and psychoemotional disorders, physical examination for the presence of TMD, and interview and physical examination for the presence of sleep disorders with particular emphasis on grinding of teeth at night and, if possible, confirmed by partner. In addition, each patient was subjected to physical extra- and intraoral examination for an accurate assessment of the condition of teeth and oral mucosa in terms of symptoms indicating the presence of bruxism and with the use of Tooth Wear Index developed by Smith and Knight. After verification of the identified symptoms in accordance with the guidelines of ICD-10-CM, the diagnosis of bruxism was carried out.

#### Inclusion Criteria

Criteria for inclusion of a patient for further examination were as follows: age above 18 years, lack of severe systemic (including genetic) diseases, lack of severe mental illness and significant mental (including genetic) disabilities, positive diagnosis of SB based on the criteria of ICD-10-CM, lack of contraindications for polysomnographic examination, and consent to participate in the study.

#### Exclusion Criteria

The exclusion criteria except inverse to inclusion included: the presence of SB caused by a diagnosed disorder or as a side effect to intake of a drug or medication; use of medicines that significantly affect the function of the nervous and muscular systems.

### Video-Polysomnography

A one-night polysomnographic examination with video recording was carried out in each of the included patients using Nox A1 (Nox Medical, Iceland) in the Sleep Laboratory at the Wroclaw Medical University. Examination took place between 10.00 p.m. and 6.00 a.m., taking into account the preferences and sleeping habits of the patient. Before the tests, the electrodes were arranged in a standard manner as recommended by the manufacturer. A modification relative to the standard distribution of electrodes included placing only bipolar leads for electromyographic recording from the masseter muscles. In agreement with the manufacturer's recommendations, a modification was made by placing the electromyographic electrodes on both sides of the origin and insertion of masseter muscles.

Each of the polysomnography examinations included electroencephalography, electrocardiography, electrooculography, and electromyography from the chin area and bilaterally from the regions of the masseter muscles, motion recording of abdominal and thoracic breathing activity, assessment of body position, as well as audio and video recording. An additional recording tool, NONIN WristOx2 3150 pulse oximeter (Nonin Medical, Inc., USA), was used which enabled the recording of the level of saturation, pulse, and plethysmographic data. The restoration of the full polysomnographic record was possible, thanks to the device Noxturnal developed for sleep recording and analysis (Nox Medical, Iceland).

**Figure 1:**
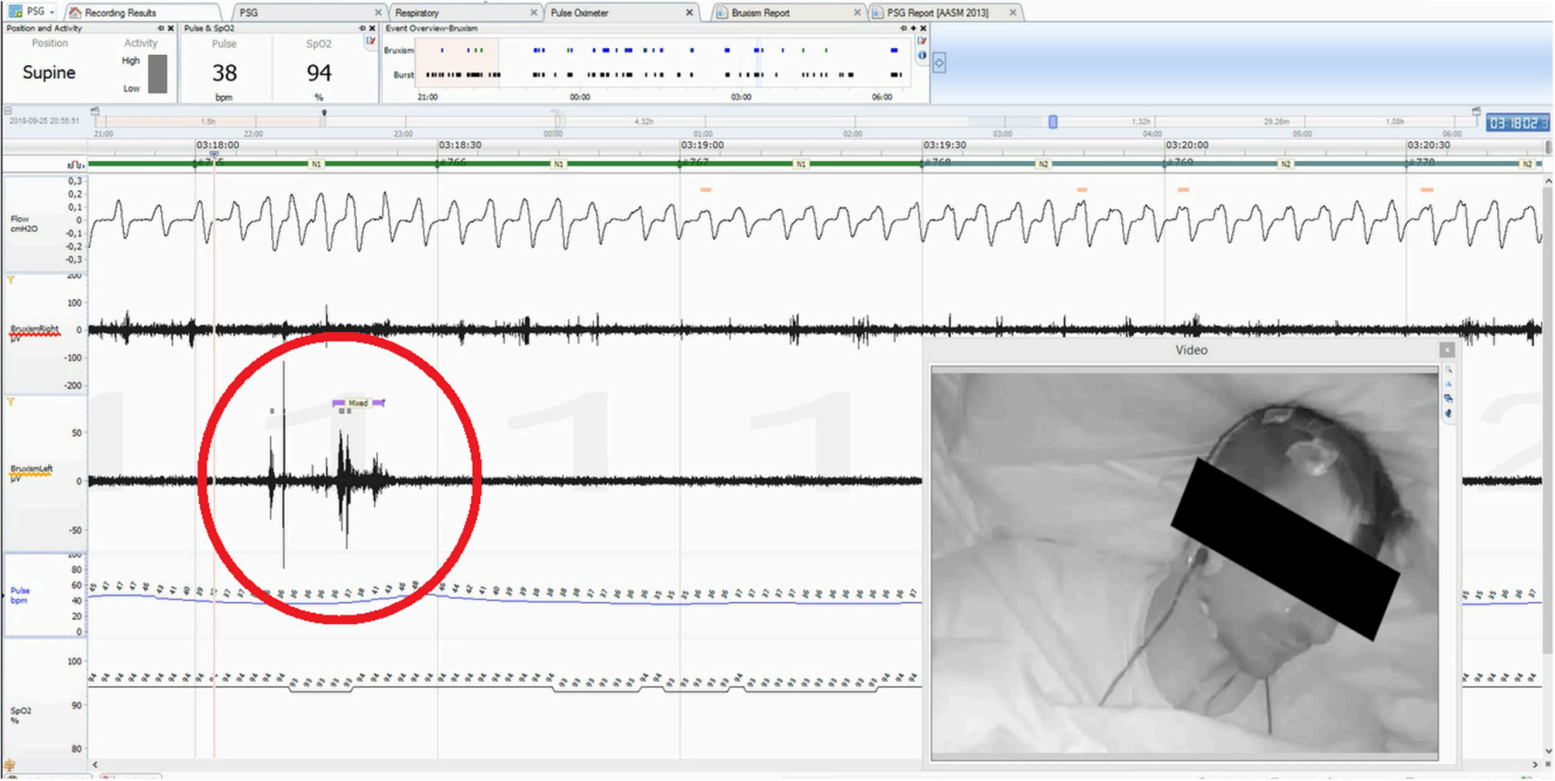
Noxturnal interface, mixed bruxism episode indicated with red circle.

Bruxism was assessed according to the ICD-10-CM guidelines based on the electromyographic recording of masseter muscles and audio and video recordings. Episodes of rhythmic activity of masseter muscles, which were often accompanied by grinding sounds and characteristic movements in the orofacial region occurring after a minimum of 3 s break from the last muscle activity, were qualified as episodes of bruxism. Based on the types of contractions, episodes were classified as phasic (lasting 0.25–2 s), tonic (lasting more than 2 s), or mixed (Figure 1).

The participants were divided into study group (bruxers—bruxism episode index (BEI) ≥ 2) and control group (non-bruxers—BEI < 2) according to the BEI (6).

### TMD Pain Screener

Each of the qualified participants was asked to fill in TMD Pain Screener. This questionnaire has been validated by the International Network for Orofacial Pain and Related Disorders Methodology (https://ubwp.buffalo.edu/rdc-tmdinternationa). It allows an easy assessment of the occurrence of pain in the temporomandibular area in patients over the last month. The survey consists of three questions. Questions 1, 2, and 3a are called as “short screener,” while questions 3b−3d are called as “long screener”. Answers are scored as follows: “a”−0 point, “b”−1 point, and “c”−2 points. The maximum number of points given for a “short screener” is 4, while for a “long screener” it is 7. The questionnaire also allows screening of pain associated with TMD.

### Database

After obtaining data from extra- and intraoral examination, polysomnography, laboratory tests, and questionnaires, a database was created in Excel (Microsoft Office, USA). Selected elements of the database were then subjected to statistical analysis.

### Statistical Analysis

A statistical analysis of the obtained data was carried out using the statistic program Statistica 13.1 (Statsoft, Poland). The level of statistical significance was assumed at α = 0.05, i.e., the results of statistical tests that appeared with a probability of *p* < 0.05 were considered statistically significant. The shapes of the data distributions and deviations from the shape of the normal distribution were analyzed with the Shapiro–Wilk test.

The following principle was adopted in the analyses: first, the use of parametric methods was preferred. If the data did not meet the assumptions of the parametric methods (e.g., due to the shapes of the distribution), they were further transformed. If the data met the assumptions after the transformation, parametric methods were used for the analyses. If the data still did not meet the assumptions after the transformation, non-parametric methods were used for the analyses, and the analyses were performed on the original (untransformed) data.

In additional analyses, the determined BEI was divided into two groups: BEI value up to 2 (“ <2”) and BEI value of 2 and above (“2+”), corresponding to the control and test group, respectively. The preferred analytical approach for the study of differences between the groups was Student's *t*-test for unrelated samples. This test verifies the hypothesis that there are no differences between means in the compared groups.

The assumptions of Student's *t*-test are as follows:

- distributions of data in the compared populations are normal, and
- in both the compared populations, there is the same variance (variances are homogeneous).

If the assumption regarding homogeneity of variance was not met, the analysis of significance of differences between means in both the groups was carried out using Cochran–Cox test. In the case of a significant breach of the assumption about the normality of the data distribution, the Mann–Whitney *U* test was used to analyze the differences between groups. This test is a non-parametric equivalent of the Student's *t*-test and verifies the hypothesis that the medians in both the compared groups are not equal or, in other words, that two randomly selected samples come from the same population. For some comparisons, the size of groups differed from the size of the starting study and control group due to incorrect or incomplete filling of the questionnaires by the patients.

## Results

### Sample Characteristics

There were 77 patients included in the study (56 women and 21 men). All the patients subjected to polysomnography were Caucasians, aged 18–63 (mean age 34.8 ± 10.8). Of the 77 patients, 58 were included in the studied group and 17 in the control group.

### BEI and Type of Electromyographic Pathway

#### Phasic Episodes

The distribution of BEI data deviated from the normal distribution (*W* = 0.8745, *p* < 0.0001). Therefore, the BEI values were subjected to a logarithmic transformation, after which the data distribution was not found to differ significantly from the normal distribution (*W* = 0.9728, *p* = 0.10). Similarly, the distribution of data of phase contraction differed statistically significantly from the normal distribution (*W* = 0.7021, *p* < 0.0001). However, even after applying the logarithmic transformation, the data distribution was found to deviate from the normal distribution (*W* = 0.7759, *p* < 0.0001).

**Figure 2:**
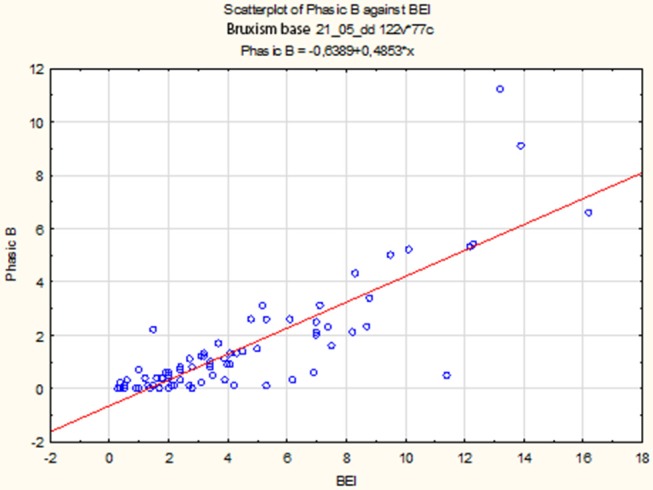
Relationship between BEI values and the number of phasic episodes.

The non-parametric Spearman rank correlation test was used to analyze the strength and significance of the BEI and the number of phasic episodes. The analysis showed a statistically significant relationship between BEI and the number of phasic episodes [*r*s_(77)_ = 0.79, *p* < 0.00001]. The increase in BEI was accompanied by an increase in the number of phasic episodes (Figure 2).

Descriptive statistics for phasic episodes are presented in Table 1.

**Table 1:** Descriptive statistics for phasic episodes in studied and control group.

	**Phasic episodes**					
	**Number**	**Average**	**Median**	**Minimum**	**Maximum**	**Standard deviation**
BEI <2	19	0.29	0.10	0	2.2	0.513
BEI 2+	58	1.90	1.15	0	11.2	2.214

The distribution of data of phasic episodes in the “BEI < 2” group deviated from the normal distribution (*W* = 0.5933, *p* < 0.0001). Similarly, the distribution of data of phasic episodes in the “BEI 2+” group differed statistically significantly from the normal distribution (*W* = 0.7470, *p* < 0.0001).

**Figure 3:**
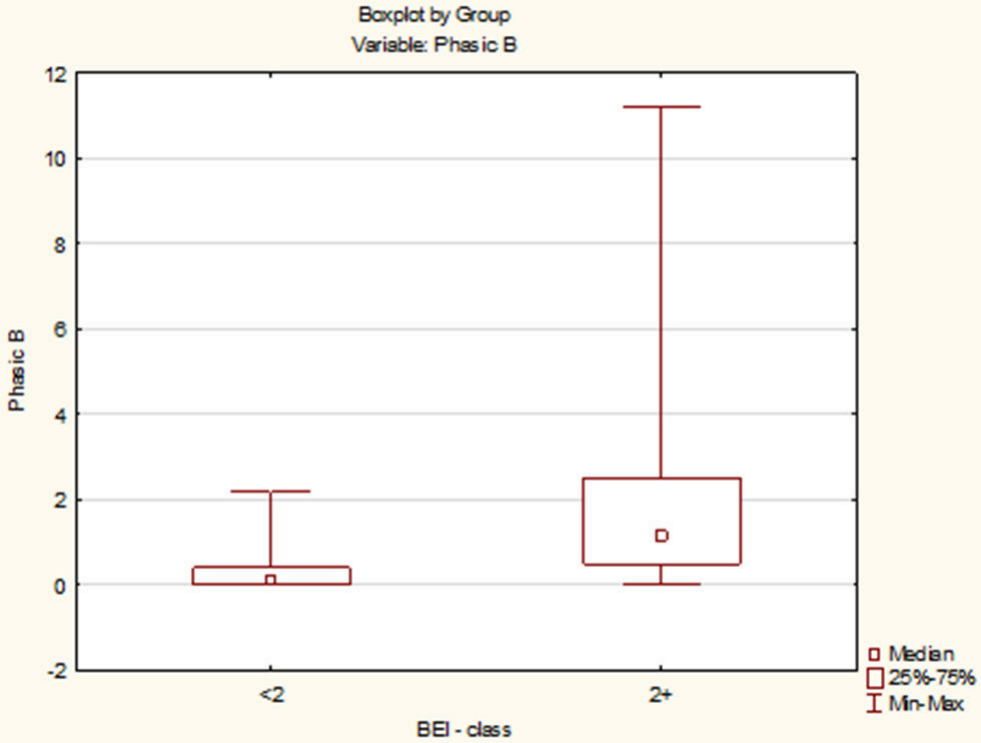
Comparison of the number of phasic episodes in the studied and control group.

Due to the violation of the assumption about the normality of the data distribution in the groups “BEI < 2” and “BEI 2+”, the Mann–Whitney *U* test was used for analysis. The analysis showed that both the groups differed significantly in terms of phasic episodes (*U* = 149.5, *p* < 0.0001), and the number of phasic episodes was statistically significantly higher in the “BEI 2+” group (Figure 3).

#### Tonic Episodes

The distribution of BEI data deviated from the normal distribution (*W* = 0.8745, *p* < 0.0001). Therefore, the BEI values were subjected to a logarithmic transformation, after which the data distribution was not found to differ significantly from the normal distribution (*W* = 0.9728, *p* = 0.10). Similarly, the distribution of data of tonic episodes differed statistically significantly from the normal distribution (*W* = 0.8858, *p* < 0.0001). However, even after applying the logarithmic transformation, the data distribution was found to deviate from the normal distribution (*W* = 0.9293, *p* = 0.0004).

**Figure 4:**
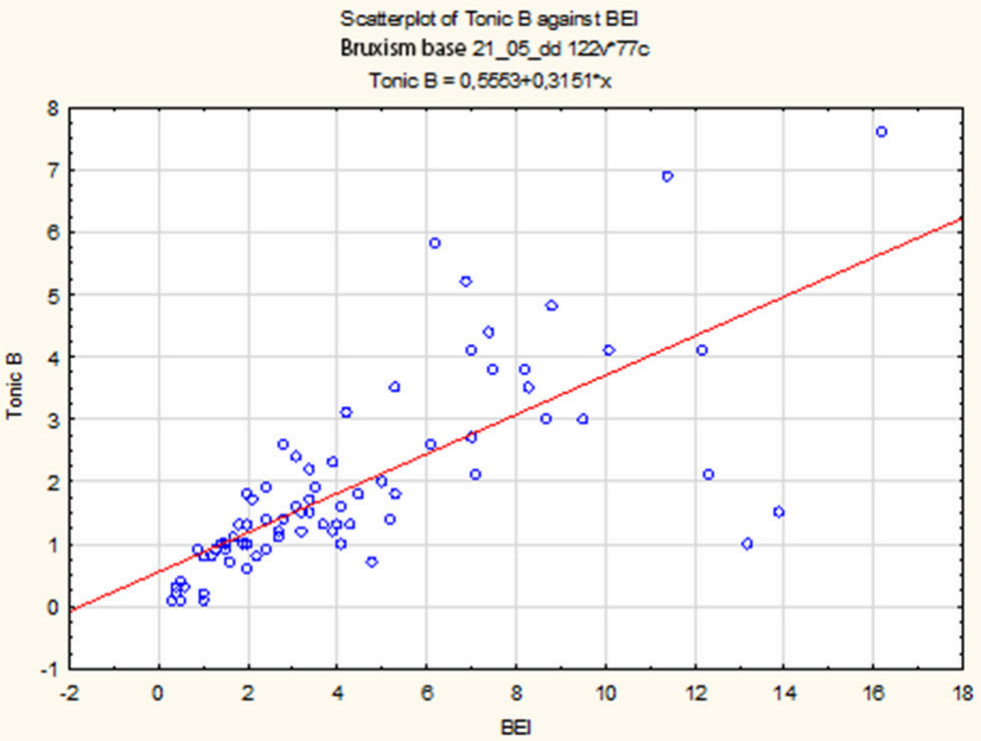
Relationship between BEI values and the number of tonic episodes.

The non-parametric Spearman rank correlation test was used to analyze the strength and significance of the BEI and the number of tonic episodes. The analysis showed a statistically significant relationship between BEI and the number of tonic episodes [*r*s_(77)_ = 0.81, *p* < 0.00001]. The increase in BEI was accompanied by an increase in the number of tonic episodes (Figure 4).

Descriptive statistics for tonic episodes are presented in Table 2.

**Table 2:** Descriptive statistics for tonic episodes in studied and control group.

	**Tonic episodes**					
	**Number**	**Average**	**Median**	**Minimum**	**Maximum**	**Standard deviation**
BEI <2	19	0.64	0.80	0.10	1.30	0.398
BEI 2+	58	2.38	1.80	0.60	7.60	1.521

The distribution of data of tonic episodes in the “BEI < 2” group deviated from the normal distribution (*W* = 0.8916, *p* = 0.03). Similarly, the distribution of data of tonic episodes in the “BEI 2+” group differed statistically significantly from the normal distribution (*W* = 0.8463, *p* < 0.0001).

**Figure 5:**
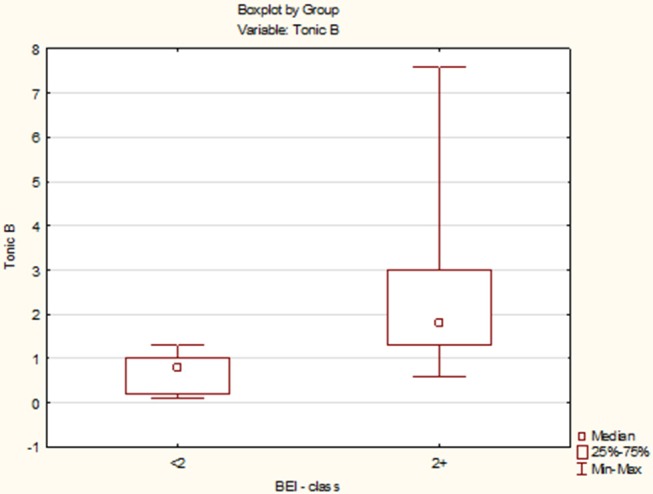
Comparison of the number of tonic episodes in the studied and control group.

Due to the violation of the assumption about the normality of the data distribution in the groups “BEI < 2” and “BEI 2+”, the Mann–Whitney *U* test was used for analysis. The analysis showed that both the groups differed significantly in terms of tonic episodes (*U* = 54.5, *p* < 0.0001), and the number of tonic episodes was statistically significantly higher in the “BEI 2+” group (Figure 5).

#### Mixed Episodes

The distribution of BEI data deviated from the normal distribution (*W* = 0.8745, *p* < 0.0001). Therefore, the BEI values were subjected to a logarithmic transformation, after which the data distribution was not found to differ significantly from the normal distribution (*W* = 0.9728, *p* = 0.10). Similarly, the distribution of data of mixed episodes differed statistically significantly from the normal distribution (*W* = 0.8386, *p* < 0.0001). However, even after applying the logarithmic transformation, the data distribution was found to deviate from the normal distribution (*W* = 0.8722, *p* < 0.0001).

**Figure 6:**
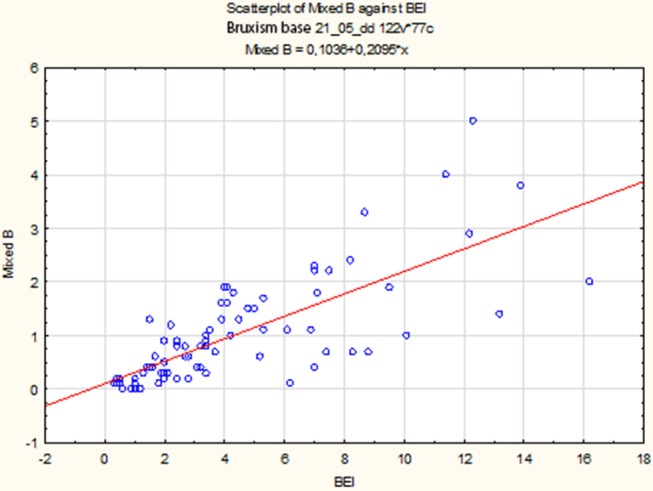
Relationship between BEI values and the number of mixed episodes.

The non-parametric Spearman rank correlation test was used to analyze the strength and significance of the BEI and the number of mixed episodes. The analysis showed a statistically significant relationship between BEI and the number of mixed contractions [*r*s_(77)_ = 0.77, *p* < 0.00001]. The increase in BEI was accompanied by an increase in the number of mixed episodes (Figure 6).

Descriptive statistics for mixed episodes are shown in Table 3.

**Table 3:** Descriptive statistics for mixed episodes in studied and control group.

	**Mixed episodes**					
	**Number**	**Average**	**Median**	**Minumum**	**Maximum**	**Standard deviation**
BEI <2	19	0.253	0.20	0	1.30	0.304
BEI 2+	58	1.283	1.00	0.10	5.00	1.008

The distribution of data of mixed episodes in the “BEI < 2” group deviated from the normal distribution (*W* = 0.7339, *p* = 0.0002). Similarly, the distribution of data of mixed episodes in the “BEI 2+” group differed statistically significantly from the normal distribution (*W* = 0.8589, *p* = 0.00001).

**Figure 7:**
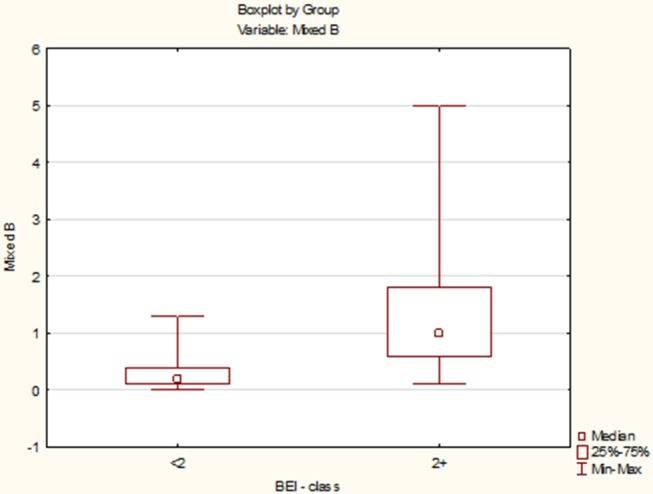
Comparison of the number of mixed episodes in the studied and control group.

Due to the violation of the assumption about the normality of the data distribution in the group “BEI < 2” and “BEI 2+”, the Mann–Whitney *U* test was used for analysis. The analysis showed that both the groups differed statistically significantly in terms of the number of mixed episodes (*U* = 102.5, *p* < 0.0001), and the number of mixed episodes was statistically significantly higher in the “BEI 2+” group (Figure 7).

### BEI and TMD Pain Screener

The distribution of BEI data deviated from the normal distribution (*W* = 0.8745, *p* < 0.0001). Therefore, the BEI values were subjected to a logarithmic transformation, after which the data distribution was not found to differ significantly from the normal distribution (*W* = 0.9728, *p* = 0.10).

**Figure 8:**
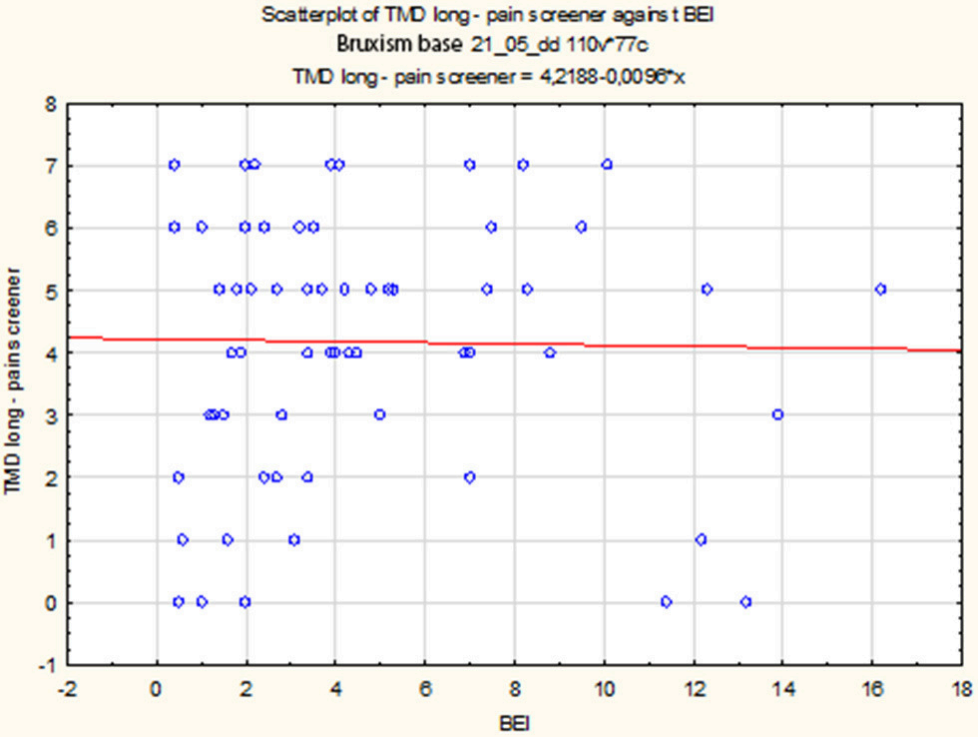
Relationship between BEI and scores of TMD Pain Screener.

The data distribution of TMD Pain Screener differed from the normal distribution (*W* = 0.9196, *p* = 0.0005). Therefore, the data of TMD Pain Screener were subjected to a second-degree power transformation. Due to the fact that the raw data of TMD Pain Screener contained 0 values, each value was increased by a fixed value 10 before the data were raised to power. However, even after applying the transformation, the data distribution was found to significantly deviate from the normal distribution (*W* = 0.9345, *p* = 0.002). The non-parametric Spearman rank correlation test was used to analyze the strength and significance of the BEI and the scores of TMD Pain Screener. The analysis showed no significant relationship between BEI and TMD Pain Screener [*r*s_(63)_ = 0.08, *p* = 0.55] (Figure 8).

Descriptive statistics for the scores of TMD Pain Screener, in patients divided into studied group and control group, are presented in the Table 4.

**Table 4:** Descriptive statistics for TMD pain screener in studied and control group.

	**TMD pain screener**					
	**Number**	**Average**	**Median**	**Minimum**	**Maximum**	**Standard deviation**
BEI <2	15	3.33	3.00	0	7	2.23
BEI 2+	48	4.44	5.00	0	7	1.98

**Figure 9:**
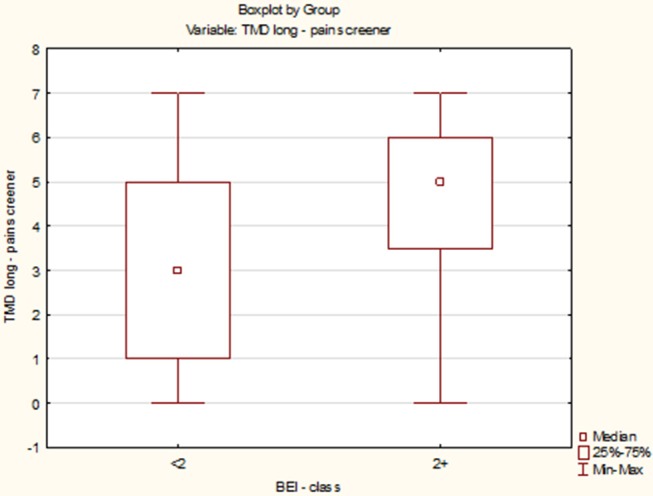
Comparison of the scores of TMD Pain Screener in studied and control group.

The distribution of data of TMD Pain Screener in the “BEI < 2” group did not differ from the normal distribution (*W* = 0.9521, *p* = 0.56). In contrast, the distribution of data of TMD Pain Screener in the “BEI 2+” group differed statistically significantly from the normal distribution (*W* = 0.9090, *p* = 0.001). Due to the violation of the assumption about the normality of the data distribution in the “BEI 2+” group, the Mann–Whitney *U* test was used for analysis. The analysis showed that both the groups did not differ significantly in terms of the results of TMD Pain Screener (*U* = 253.0, *p* = 0.08) (Figure 9).

## Discussion

Presented study showed that increase in BEI is associated with increased number of all types of muscle contractions. The number of phasic, tonic and mixed episodes was statistically significantly higher in bruxers than non-bruxers. Authors used TMD Pain Screener to assess the relationship between the intensity of SB and occurrence of pain related to TMD. Analysis showed no statistically significant relationship between intensity of SB and TMD Pain Screener scores taking into account all studied material and when dividing it into studied and control group. Summarizing, study showed that intensity of bruxism is associated to muscle activity, but not to pain related to TMD.

Bruxism during sleep is inextricably linked to the function of the masticatory muscles in two ways: it is caused by the increased activity of muscles, or it acts as a possible important risk factor for the occurrence and severity of pain related to TMD, the essential component of which are the disorders of muscle origin. The consequences of bruxism during sleep related to muscle pain are very common and sometimes severe (1–3, 13–16).

Lobbezoo et al. indicate that the possible negative clinical implications of bruxism are often related to the type of electromyographic activity, and not the number of episodes (2). Other authors also indicate this assertion (17). BEI represents only the number of bruxism episodes per hour of sleep without taking into account their duration and strength, what can be crucial for development of serious clinical consequences. In this case, the BEI may turn out to be an unreliable factor, and therefore, we should consider not only the intensification of muscle activity but also its character. It may be useful in this case to look at the type of dominating episode. However, it should be remembered that an increase in the number of episodes of bruxism per hour of sleep is always accompanied by an increase in the number of overall contractions, as well as an increase in each type of contractions separately.

In the present study, the authors verified the activity of masticatory muscles during sleep both quantitatively and qualitatively. BEI was used in the quantitative assessment, and the classification of episodes as phasic, tonic, and mixed was applied in qualitative assessment. The analysis showed that the increase of BEI was statistically significantly correlated with increase in the number of all types of electromyographic pathways, and thus, the types of episodes. This result suggests that increase in specific type of muscle activity could be associated with more frequent conditions contributing to pain related to TMD.

Many scientific teams have investigated the relationship between the comorbidity of TMD and bruxism. Blanco Aguilera et al. attempted to determine the correlation between SB and pain in temporomandibular region based on the sensitivity, age, and gender of patients and clinical subtypes of TMD. They reported that the occurrence of bruxism during sleep correlated positively with age below 60, female sex, more intensification of pain symptoms, and muscular as well as articular TMD. The study supports the hypothesis of bruxism as a risk factor for more frequent TMD. However, the study does not indicate a more frequent occurrence of the muscular disorders than the articular disorders (18). In a clinical trial of the coexistence of bruxism and TMD, Kapusevska et al. found that proper management of bruxism leads to a significant reduction in the number and intensity of symptoms of both the joint component and the muscular TMD (19). In addition, in a clinical trial determining the role of bruxism as a risk factor in the formation and exacerbation of TMD, Sierwald et al. observed almost the same share of SB and AB. They also found a significant increase in the risk of occurrence of TMD in the case of patients with both types of bruxism (20). Raphael et al. aimed to evaluate sleep background electromyographic activity remaining after activity attributable to SB with removing other orofacial activity, other oromotor activity and movement artifacts in women suffering from chronic myofascial TMD. Results of this study indicated that background electromyographic activity during non-sleep bruxism event periods was higher in women with myofascial TMD. Background electromyographic activity in contrast to sleep bruxism event-related activity was also associated with pain intensity. This study indicates the need for a broader view of muscle activity during sleep (21).

In the present work, to examine the relationship between the intensity of SB and the occurrence of pain caused by TMD, TMD Pain Screener, a questionnaire validated by the International Network for Orofacial Pain and Related Disorders Methodology, was used (12). TMD Pain Screener is used to examine the occurrence of pain in the jaw and temple area (22). Statistical analysis showed that higher values of BEI did not correlate with higher scores of the TMD Pain Screener. In addition, more severe bruxism was not found to correlate with more severe pain in temporomandibular area, even if increased number of all types of muscular activities was taken into account. This observation may be due to the inaccuracy of diagnostic methods which are based only on patients' observations, and in all conditions, they should be compared with the results of the clinical examination. TMD Pain Screener could be of great diagnostic significance as a tool for assessing pain associated with TMD, but maybe not in the context of pain associated with TMD caused by SB because this relationship was not supported in this study.

Berger et al. conducted a study analyzing the association between TMD-related pain and specific diagnoses of bruxism, both based on questionnaires. Patients were asked to fill an anonymous questionnaire, consisting of three questions, to verify the presence of TMD-related pain and the two forms of bruxism. In this study, the authors also showed that there was no statistically significant association between SB and TMD-related pain (23). A major limitation of this study was that the diagnosis of both bruxism and pain was based only on questionnaires. Also, van Selms et al. investigated whether pain related to TMD is caused by an interaction between psychological factors and self-reported bruxism activities. Patients were diagnosed with TMD-related pain according to the Diagnostic Criteria for Temporomandibular Disorders (DC/TMD). The authors also found that there were no significant interactions between any of the psychological factors and bruxism with respect to the clinical presence of TMD-related pain (24). A strength of this study was the application of DC/TMD to determine pain, but bruxism was diagnosed only on the basis of a questionnaire, which makes it impossible to arrive at a conclusion. On the other hand, Reissmann et al. conducted a study exploring whether self-reported awake and SB interact in their associations with painful TMD (assessed in accordance with DC/TMD) and whether the interaction is multiplicative or additive. The authors found that awake and SB are associated with an increased presence of painful TMD, and that both types of bruxism are not independently associated, but interact additively (25). Again, a limitation of the study was the use of self-reporting to determine the occurrence of bruxism. It should also be noted that some studies consider both awake and SB. In the light of the latest, separate definitions of these behaviors, it is possible that only the coexistence of both types of bruxism has an effect on the appearance of pain symptoms. However, this assumption should be studied more thoroughly.

The limitation of the study was lack of adaptive night and providing only one-night polysomnography. The study was conducted in such way due to the lack of foundation of the adaptive night by the Polish National Health Service.

## Conclusions

The occurrence of TMD-related pain is not related to the intensity of SB. TMD Pain Screener may be used as an auxiliary tool in the diagnosis or risk of occurrence of pain related to TMD, whereas in the case of SB, it has only limited diagnostic value.

## Author Contributions

JS analyzed the data and wrote the manuscript. HM and MW created the research concept, edited the manuscript, and finally revised it before submission. MM-Z recruited patients for the study and collected data. AW collected the references. GM and EW finally revised the manuscript before submission. All authors read and approved the final manuscript.

### Conflict of Interest Statement

The authors declare that the research was conducted in the absence of any commercial or financial relationships that could be construed as a potential conflict of interest.
